# Isocycloseram susceptibility baseline, and control failure likelihood in *Leucoptera coffeella* (Lepidoptera: Lyonetiidae)

**DOI:** 10.1093/jisesa/ieag032

**Published:** 2026-05-12

**Authors:** Ryan Fernando Silva e Silva, Carlos Gustavo da Cruz, Lariça Ferreira Silva, Maria Elisa de Sena Fernandes, Flávio Lemes Fernandes

**Affiliations:** Instituto de Ciências Agrárias, Universidade Federal de Viçosa, Rio Paranaíba, MG, Brazil; Instituto de Ciências Agrárias, Universidade Federal de Viçosa, Rio Paranaíba, MG, Brazil; Instituto de Ciências Agrárias, Universidade Federal de Viçosa, Rio Paranaíba, MG, Brazil; Instituto de Ciências Agrárias, Universidade Federal de Viçosa, Rio Paranaíba, MG, Brazil; Instituto de Ciências Agrárias, Universidade Federal de Viçosa, Rio Paranaíba, MG, Brazil

**Keywords:** resistant, isoxazoline, coffee, baseline susceptibility

## Abstract

The coffee leaf miner is a key pest of coffee in Brazil, controlled mostly by chemical insecticides. The recent introduction of isocycloseram (group 30) requires baseline susceptibility data to support resistance management programs. This study established the baseline susceptibility and monitored the resistance of coffee leaf miner populations to isocycloseram in the main coffee-producing regions of Brazil. Fourteen field populations were collected between June and October 2024 for the baseline assays, while 22 populations were monitored until 2024 and 2025. The bioassays revealed significant geographic variation in susceptibility, with lethal concentration LC_50_ values ranging 7.5-fold (0.95 to 7.11 mg a.i. L^−1^). Despite this, all populations remained susceptible, with resistance ratio (RR_50_) ≤ 1.0, indicating absence of preexisting resistance. The diagnostic concentration bioassays showed consistently high mortality (>90%), confirming continuous susceptibility. Efficacy trials with the label dose (150 mg a.i. L^−1^) resulted in mortality between 93% and 100%, with negligible probability of control failure. The diagnostic concentrations (LC_99_ = 39.76 mg a.i. L^−1^) may be used in future monitoring. The monitoring and the adoption of integrated resistance management strategies are fundamental to preserve the long-term efficacy of this insecticide.

## Introduction

The judicious use of insecticides in agriculture is one of the main challenges of modern agricultural production, especially in perennial crops like coffee, where pest management must be continuous and effective across several crop seasons (Merhi et al. 2022, Misra and Yadav 2023). Despite the growing adoption of more sustainable approaches, such as Integrated Pest Management, chemical control remains the most widely used tactic, especially against pests of major economic importance (Zhou et al. 2024, Lazarević-Pašti et al. 2025). In this context, the introduction of new active ingredients with different modes of action is crucial to mitigate resistance selection and preserve the efficacy of the active ingredients used (Sparks and Bryant 2022, Jeschke 2025).

In coffee production systems, chemical control programs in recent decades have relied heavily on organophosphates, neonicotinoids and, more recently, diamides, increasing selection pressure and contributing to the evolution of resistant pest populations (Leite et al. 2020, 2021). The difficulty in developing new molecules with innovative modes of action has restricted the diversification of options available on the market. Between 1990 and 2020, only a few new chemical classes were successfully introduced, notably diamides and, more recently, isoxazolines (Gonçalves et al. 2021, Jeschke 2024, Kong et al. 2025). This slow pace of innovation reinforces concerns about resistance evolution and the sustainability of current chemical control strategies (Araújo et al. 2023).

Isoxazolines represent a new generation of neurotoxic insecticides that act as negative allosteric modulators of GABA (gamma-aminobutyric acid)-gated chloride channels, particularly on the RDL (resistant to dieldrin) receptor, causing neuronal depolarization and insect death (Kong et al. 2025). Isocycloseram, marketed as Joiner (Syngenta), is a compound from this class that exhibits a broad spectrum of activity, low mammalian toxicity, and moderate selectivity for natural enemies (Cassayre et al. 2021, Bordini et al. 2025). Available on the global market since 2021, its use is still considered recent, especially in crops like coffee. In Brazil, the registration of isocycloseram is even more recent, being approved only in 2023 (MAPA 2023), which implies a lack of historical data regarding the efficacy and susceptibility of the coffee leaf miner, *Leucoptera coffeella* (Guérin-Méneville) (Lepidoptera: Lyonetiidae), to this compound. In this context, it is necessary to adopt measures to preserve the efficacy of isocycloseram for a longer period, thereby extending the longevity of the technology in the management of *L. coffeella*. Therefore, the immediate implementation of baseline susceptibility studies is required before the molecule is applied intensively and widely across Brazil. Baseline susceptibility studies involve collecting insect populations from different production regions, performing toxicity bioassays, and determining dose–response curves to obtain diagnostic lethal doses for 50% (LD_50_), 80% (LD_80_), and 99% (LD_99_) mortality. These diagnostic doses are essential for resistance monitoring studies, allowing the tracking of resistance evolution in insect populations and supporting informed decisions in insecticide management (Roush and Miller 1986, Ffrench-Constant and Roush 1990, Costa et al. 2020). Although these doses can be determined, the LC_99_ is the most commonly used for early detection of resistance, providing sufficient time to implement resistance management programs (WHO 2016).

The increasing expansion of agricultural use of isocycloseram, especially in tropical crops like coffee, raises questions about its medium- and long-term sustainability, considering the imminent risk of resistance evolution. The coffee leaf miner is one of the main threats to coffee cultivation in Brazil, responsible for significant losses in productivity and quality (Dantas et al. 2021). Its short biological cycle, high reproduction rate, and occurrence of multiple generations per year favor the rapid selection of resistant individuals in populations repeatedly exposed to insecticides (Leite et al. 2020). Populations of *L. coffeella* resistant to different insecticide classes, notably organophosphates, neonicotinoids, and even recent reports involving diamides (chlorantraniliprole), have already been recorded in major coffee-growing regions of Brazil (Leite et al. 2020, 2021, Souza et al. 2025).

Control failures caused by resistant populations can substantially increase management costs, particularly in regions such as the Brazilian Cerrado, where expenses may reach US$247.70 per hectare, with most of this value attributed to insecticides (Picanço Filho et al. 2024). Although isocycloseram is a recently introduced molecule, previous experiences with other insecticide classes in coffee demonstrate that resistance can evolve under intensive use (Leite et al. 2020, 2021). Moreover, once established, resistance tends to decline slowly and unpredictably due to continued selection pressure (Rocha et al. 2022). Therefore, early establishment of baseline susceptibility is essential to enable proactive resistance monitoring and delay potential control failures.

Given the above, especially for new molecules like isocycloseram, it is essential to establish baseline curves through concentration-mortality bioassays conducted on various populations from different coffee-growing regions of Brazil. These curves allow for the definition of diagnostic concentrations and the early detection of changes in pest susceptibility, facilitating preventive resistance management. Thus, this study aims to determine the baseline susceptibility curves of Brazilian populations of the coffee leaf miner to isocycloseram, supporting effective strategies to ensure the sustainable use of this new molecule in coffee cultivation. In addition, we determined the control failure likelihood (CFL) to verify whether the populations are truly sensitive to isocycloseram, since this insecticide was introduced in Brazil in 2023 and control failures could be present.

## Materials and Methods

The study was conducted at the Entomology Laboratory—*Campus* Rio Paranaíba (UFV-CRP), Minas Gerais State, Brazil. Population sampling was carried out in major coffee-growing regions of Brazil, predominantly located within the Brazilian Cerrado biome. These areas were prioritized due to climatic conditions (hot and dry), which favor rapid development cycles of the coffee leaf miner—as short as 11 d during the hottest and driest period (Giraldo-Jaramillo et al. 2024)—and a history of frequent insecticide applications, which exerts a high selection pressure for insecticide resistance (Rocha et al. 2022).

### Population Sampling

**Fig. 1.**
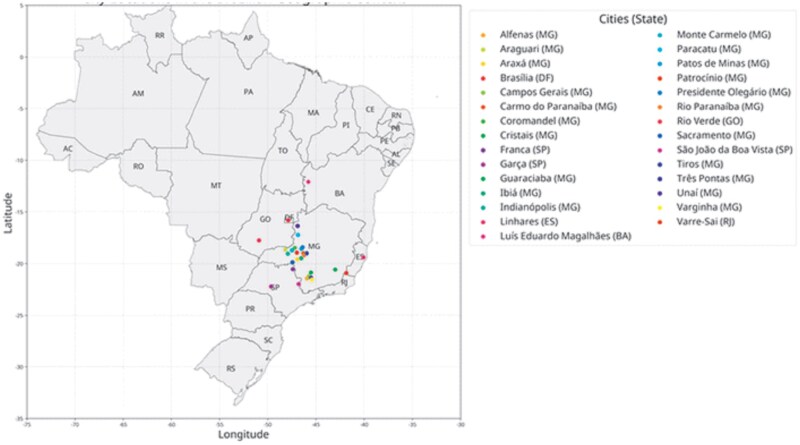
Geographic distribution of Brazilian municipalities where *L. coffeella* populations were collected.

Leaves from plagiotropic branches bearing active mines were collected from commercial coffee farms in Brazil (Fig. 1). Approximately 800 to 1,000 leaves were collected, totaling around 2,400 ± 150.45 insects per population. The collected leaves were stored in 15-kg brown Kraft paper bags (52 × 24 × 6 cm) and transported to the Entomology Laboratory of UFV-CRP for baseline studies.

For the baseline study, 14 populations of *L. coffeella* were collected between June and October 2024 from major coffee-growing regions of Brazil. In 2024 and 2025, over 22 populations per year from the same regions were evaluated for mortality using the label rate of isocycloseram (Table 1 and Fig. 1).

**Table 1.** Sampling sites and their geographical coordinates for the field populations used in the study for the coffee leaf miner *L. coffeella*

Municipality	Coordinates	Municipality	Coordinates	Municipality	Coordinates
**Araguari 1-MG**	18°36ʹ38″S; 48°11ʹ58″W	Patos de Minas-MG	18°34ʹ21″S; 46°30ʹ59″W	Varginha-MG	19°12ʹ27″S; 46°30ʹ23″W
**Araguari 2-MG**	18°35ʹ32.52″S; 48°11ʹ12.33″W	Patrocínio 1-MG	18°56ʹ11.81″S; 46°56ʹ52.11″W	São João da Boa Vista 1-SP	23°54ʹ23″S; 49°39ʹ12″W
**Araxá-MG**	19°35ʹ17″S; 46°56ʹ39″W	Patrocínio 2-MG	18°58ʹ46.76″S; 46°57ʹ16.38″W	São João da Boa Vista 2-SP	21°58ʹ5.33″S; 46°51ʹ31.44″W
**Campos Gerais-MG**	21°14ʹ15″S; 45°45ʹ29″W	Patrocínio 3-MG	18°58ʹ7.83″S; 46°56ʹ3.88″W	Franca-SP	20°31ʹ50″S; 47°24ʹ06″W
**Carmo do Paranaíba-MG**	19°00ʹ03″S; 46°18ʹ33″W	Patrocínio 4-MG	18°59ʹ51.74″S; 46°54ʹ20.19″W	Franca-SP	20°31ʹ50″S; 47°24ʹ06″W
**Coromandel 1-MG**	18°12ʹ47″S; 47°07ʹ29″W	Presidente Olegário 1-MG	18°22ʹ39″S; 46°25ʹ08″W	Linhares-ES	19°23ʹ09″S; 40°03ʹ59″W
**Coromandel 2-MG**	18°35ʹ47.46″S; 46°55ʹ8.89″W	Presidente Olegário 2-MG	18°22ʹ20.55″S; 46°29ʹ8.87″W	Rio Verde-GO	18°7ʹ45.37″S; 50°43ʹ52.60″W
**Coromandel 3-MG**	18°35ʹ34.36″S; 46°54ʹ16.22″W	Presidente Olegário 3-MG	18°17ʹ56.52″S; 46°33ʹ10.71″W	Brasília-DF	15°56ʹ34″S; 47°56ʹ28″W
**Coromandel 4-MG**	18°36ʹ14.78″S; 46°53ʹ19.44″W	Rio Paranaíba 1-MG	19°12ʹ27″S; 46°30ʹ23″W	Varre-Sai-RJ	20°57ʹ23″S; 41°54ʹ15″W
**Coromandel 5-MG**	18°35ʹ14.30″S; 46°53ʹ35.11″W	Rio Paranaíba 2-MG	19°12ʹ34.96″S; 46°13ʹ21.01″W	Luís Eduardo Magalhães-BA	12°03ʹ54″S; 45°54ʹ12″W
**Cristais-MG**	20°51ʹ19″S; 45°31ʹ07″W	Rio Paranaíba 3-MG	19°13ʹ1.47″S; 46°13ʹ8.91″W	Acronym/States from Brazil	
**Guaraciaba-MG**	20°34ʹ03″S; 43°00ʹ23″W	Rio Paranaíba 4-MG	19°12ʹ59.33″S; 46°13ʹ57.21″W	MG = Minas Gerais	BA = Bahia
**Ibiá-MG**	19°29ʹ07″S; 46°32ʹ38″W	Rio Paranaíba 5-MG	19°10ʹ14.31″S; 46°12ʹ52.19″W	SP = São Paulo	GO = Goiás
**Indianópolis-MG**	19°01ʹ35″S; 48°04ʹ06″W	Sacramento-MG	19°51ʹ09″S; 47°26ʹ34″W	ES = Espírito Santo	DF = Distrito Federal
**Monte Carmelo 1-MG**	18°42ʹ21″S; 47°30ʹ18″W	Tiros-MG	19°00ʹ56″S; 45°57ʹ42″W	RJ = Rio de Janeiro	
**Monte Carmelo 2-MG**	18°41ʹ35.49″S; 47°32ʹ28.08″W	Três Pontas-MG	21°21ʹ34″S; 45°30ʹ36″W		
**Paracatu-MG**	17°10ʹ32″S; 46°52ʹ18″W	Unaí-MG	16°19ʹ31″S; 46°54ʹ21″W		

### Insect Rearing

Mined leaves from the field-collected populations were sent to the Entomology Laboratory of UFV-CRP. Leaves with predated mines, old mines, or signs of parasitism were discarded. After selection, leaves from each location were maintained in entomological cages (50 × 50 × 50 cm), constructed with aphid-proof screen and lined with cotton moistened with deionized water to prevent desiccation and promote adult emergence. Each cage was labeled with the collection site name and maintained under controlled conditions at 25 ± 1°C, 65 ± 3% relative humidity, and a 12:12 h (L:D) photoperiod.

After emergence, adults were aspirated and transferred to new entomological cages (50 × 50 × 50 cm). Four coffee seedlings of the susceptible cv Paraíso were added to each cage as a substrate for oviposition and larval feeding. Every 7 d, the seedlings were replaced with new ones to maintain the laboratory population. Adults were fed with a 10% honey solution in deionized water. This diet was provided ad libitum through a sterilized cotton wick soaked in the solution and suspended in the upper part of the cage.

### Baseline Bioassay

The baseline bioassay to determine the diagnostic doses of isocycloseram for *L. coffeella* populations was conducted following the method described by Gonring et al. (2019). In this study, 14 Brazilian populations from traditional coffee-producing regions were used (Table 1). For this purpose, the commercial insecticide Joiner 200 CS (isocycloseram 200 g L^−1^; Syngenta Ltd.) was acquired. Initially, the insecticide was diluted to obtain 3 concentrations, which were used as pretests to simulate dose–response curves. This pretest is necessary to reduce errors in selecting the final dose–response curves. Based on the results of the pretest, 13 concentrations of isocycloseram (0.5, 2, 5, 10, 25, 35, 50, 60, 80, 100, 125, 135, and 150 mg a.i. L^−1^) were tested to obtain mortality ranging from 5% to 95%. Isocycloseram was diluted in 200 ml of distilled water to prepare the stock solution.

After dilution, coffee seedlings of cv. Paraíso 2, grown in plastic bags with 6 leaf pairs and free of insecticide residues, were selected to receive the treatments. Applications were performed using a Wimpel W-77 airbrush, with a spray volume of 10 ml per plant at a pressure of 15 psi. The equipment was held at a distance of 25 cm from each seedling to ensure uniform coverage of all leaves with the same volume of solution for each treatment. After drying, the seedlings were placed in polypropylene Biocreation cages (50 × 50 × 50 cm), maintaining 4 seedlings per cage per treatment. In each cage, 60 one-day-old adults of *L. coffeella*, originating from the laboratory colony, were added. Adults were separated by population. The adults remained in the cages for 48 h and were then removed using a laboratory-made electric aspirator. This exposure period allowed the deposition of 20 to 30 eggs of *L. coffeella* per seedling. Seven days later, seedlings from each treatment were inspected for larval hatching. At this time, 20 mines per seedling were marked to assess mortality. Mines were pierced at the edges to remove newly hatched larvae, which were examined with a fine brush to determine whether they were alive or dead. The number of dead larvae per treatment, replicate, and population was converted into a percentage to calculate mortality.

The mortality results for each insecticide and population were corrected for the natural mortality observed in the control (Abbott 1925), and subjected to probit analysis using the PROBIT procedure in SAS (SAS Institute Inc. 2022). The data from all surveyed populations were pooled to obtain overall estimates and selection of suitable diagnostic concentration (LC_99_; Table 2) for future studies monitoring coffee leaf miner resistance to isocycloseram (Caballero et al. 2015). The toxicity ratio was calculated as the ratio of the estimated lethal concentration 50 to population less susceptible by population more susceptible using the formula: TR = greater LC_50_/lower LC_50_. The 95% confidence intervals for TR were determined following Robertson et al. (2017) method and were significant if intervals did not include a value of 1.

**Table 2.** Lethal concentration of isocycloseram in populations of *L. coffeella* from the major coffee-growing regions of Brazil^a^

State^b^	Population	*n*	Slope (±SE)	Concentration (95% CI) mg a.i. L⁻^1^			χ^ 2^	*P-*value	Resistance ratio [RR_50_] (95% CI)
				LC_50_	LC_80_	LC_99_			
**BA**	Luís Ed. Magalhães	800	1.29 ± 0.25	6.95 (4.57 to 15.45)	31.01 (14.31 to 43.95)	67.80 (45.50 to 88.67)	0.20 (8)	0.99	7.32 (7.10 to 7.48)^*^
**MG**	Patrocínio	960	1.03 ± 0.13	3.68 (1.92 to 6.06)	23.90 (14.66 to 44.16)	63.57 (35.58 to 95.41)	1.27 (10)	0.99	3.87 (0.89 to 4.31)
	Carmo do Paranaíba	800	0.97 ± 0.13	2.39 (1.04 to 4.23)	17.61 (10.70 to 20.88)	50.04 (28.79 to 95.28)	0.94 (8)	0.99	2.51 (0.32 to 3.00)
	Rio Paranaíba 1	1,040	1.07 ± 0.14	3.19 (1.87 to 4.92)	19.27 (12.18 to 35.70)	49.36 (27.85 to 88.74)	0.22 (11)	0.99	3.36 (0.20 to 3.61)
	Rio Paranaíba 2	800	2.33 ± 0.49	3.01 (2.46 to 3.71)	6.88 (5.11 to 13.12)	10.61 (7.05 to 26.95)	0.002 (8)	0.99	3.16 (0.23 to 4.44)
	Rio Paranaíba 3	640	1.15 ± 0.26	1.06 (0.15 to 2.41)	5.64 (2.51 to 9.49)	13.54 (7.96 to 26.33)	1.38 (6)	0.96	1.12 (0.78 to 2.08)
	Alfenas	800	0.76 ± 0.11	2.83 (1.33 to 4.75)	36.08 (21.80 to 53.69)	136.60 (68.03 to 188.2)	2.81 (8)	0.93	2.98 (0.20 to 3.46)
	Araxá	960	1.09 ± 0.04	7.11 (5.75 to 8.71)	41.94 (34.07 to 52.37)	106.04 (83.23 to 138.8)	2.35 (10)	0.99	7.48 (6.35 to 9.24)^*^
	Varginha	720	1.49 ± 0.26	3.29 (2.24 to 6.14)	11.98 (6.35 to 40.60)	23.56 (10.68 to 48.35)	0.87 (7)	0.99	3.46 (0.17 to 4.40)
**SP**	Franca	960	1.24 ± 0.18	1.08 (0.25 to 2.15)	4.55 (3.11 to 5.17)	31.57 (11.28 to 48.25)	1.42 (10)	0.99	1.14 (0.77 to 1.97)
**ES**	Linhares	720	1.35 ± 0.15	4.21 (3.02 to 5.24)	5.26 (4.15 to 5.35)	18.58 (15.25 to 30.24)	0.89 (7)	0.99	4.43 (0.15 to 0.34)
**GO**	Rio Verde	960	1.22 ± 0.01	2.34 (1.00 to 3.37)	5.00 (4.21 to 5.74)	20.05 (5.28 to 34.19)	0.16 (10)	0.99	2.46 (0.32 to 3.51)
**DF**	Brasília	880	1.07 ± 0.16	1.33 (0.11 to 2.28)	3.99 (3.15 to 4.82)	27.20 (11.28 to 39.71)	0.52 (9)	0.99	1.40 (0.58 to 1.83)
**RJ**	Varre-Sai	640	1.07 ± 0.16	0.95 (0.10 to 1.27)	3.58 (2.28 to 4.51)	23.21 (10.14 to 50.14)	0.005 (6)	0.99	1.00
**Pooled data**		11,680	0.84 ± 0.06	1.23 (0.74 to 1.81)	12.06 (9.58 to 15.09)	39.76 (30.84 to 53.90)	0.68	0.99	1.16 (0.03 to 5.87)

a Chi-square (χ^2^) with *P* > 0.05 indicates adequate fit to the probit model; *n*, number of insects/replicate; CI, confidence interval; a.i., active ingredient; LC, lethal concentration; RR_50_, resistance ratio; RR_50_, LC_50_ of tested population/LC_50_ of susceptible population. An asterisk (*) following the resistance ratio at lethal concentration (LC_50_) indicates significant difference from the standard susceptible population when the confidence interval does not include the value 1, following Robertson et al. (2017). Pooled data according to Caballero et al. (2015).

b States: BA, Bahia; MG, Minas Gerais; SP, São Paulo; ES, Espírito Santo; GO, Goiás; DF, Distrito Federal; RJ, Rio de Janeiro.

### CFL Bioassay

For the evaluation of CFL, a treatment with the label dose of isocycloseram (150 mg a.i. L^−1^; flow rate of 400 L of water ha^−1^) was used. A control treatment (water only) was included. We applied the same method of dilution, isocycloseram application, and evaluation of *L. coffeella* mortality as used in the bioassay for baseline determination. Each population was evaluated in a completely randomized experimental design with 4 replicates per treatment. After insecticide application, each seedling was infested with 60 adults of *L. coffeella*, which remained in the cages for 48 h for oviposition. After this period, the insects were removed. Mortality was corrected using Abbott’s formula (Abbott 1925). Evaluations of the larvicidal effect were conducted 1 wk after the oviposition period, recording the number of live and dead larvae. The CFL for each population was calculated using the formula: CFL = 100 − [(observed mortality × 100)/80] (Guedes 2017), considering 80% as the minimum acceptable mortality for product registration (MAPA 1995). Negative CFL values indicate a negligible risk of control failure (Guedes 2017).

### Spatial Data Interpolation

Each population collection site was georeferenced (Table 1), and the LC_99_ data were interpolated to generate spatial distribution maps. To visualize the geographical patterns of susceptibility and field efficacy, spatial interpolation was performed using the Inverse Distance Weighted method. This method estimates values for unsampled locations based on the weighted influence of surrounding known points, where the weight is inversely proportional to the distance (Shepard 1968). All interpolations were conducted in QGIS software (version 3.34.8-Prizren) using a power parameter of 2 to generate continuous distribution surfaces.

## Results

### Baseline

**Fig. 2.**
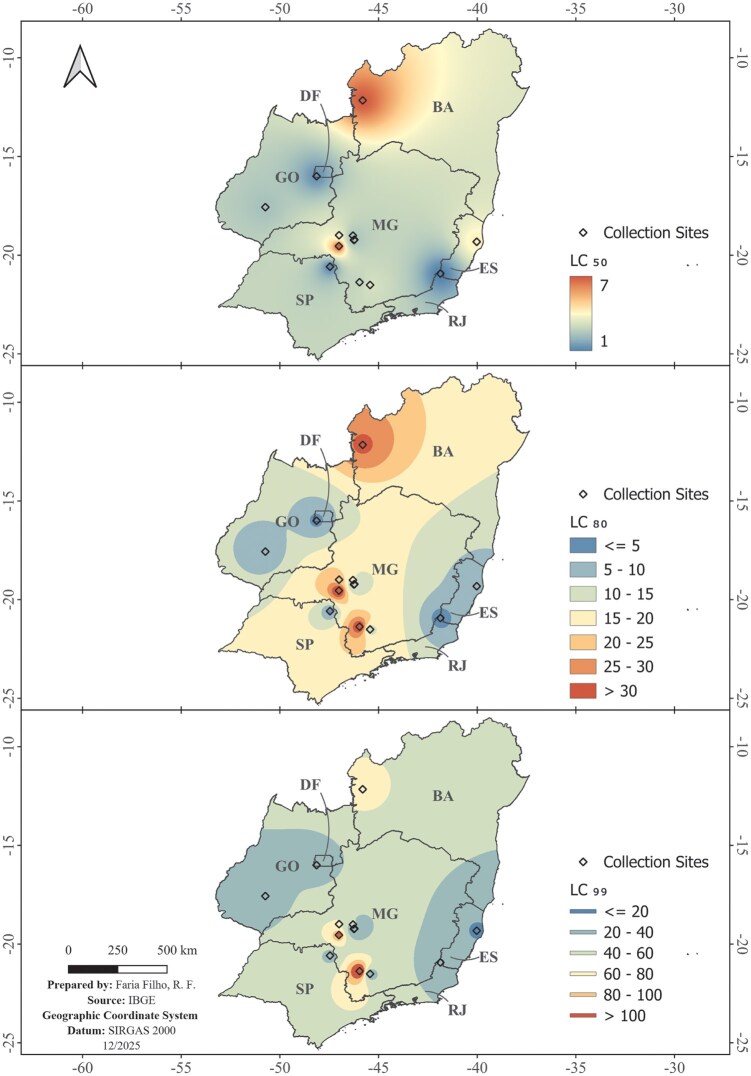
Spatial interpolation of the baseline susceptibility of *L. coffeella* populations to isocycloseram in the main coffee-producing regions of Brazil. The maps depict the geographical distribution of the median lethal concentration (LC_50_), and the concentrations lethal to 80% (LC_80_) and 99% (LC_99_) of the population, expressed in mg of active ingredient (a.i.) per liter (L^−1^). White diamond-shaped points indicate the collection sites of the populations. The color gradient (blue/green to red/yellow) indicates the susceptibility gradient (higher to lower susceptibility, respectively).

Fourteen field populations demonstrated similar susceptibility to the isocycloseram insecticide (Table 2). The median lethal concentration (LC_50_) varied approximately 7.5-fold among populations, with the highest susceptibility recorded in the Varre-Sai (RJ) population (LC_50_ = 0.95 mg a.i. L^−1^) and the lowest susceptibility in the Araxá (MG) population (LC_50_ = 7.11 mg a.i. L^−1^). The spatial distribution of this susceptibility shows a small geographical pattern (Fig. 2), where the lethal concentrations (LC_50_, LC_80_, and LC_99_) indicate that populations collected in the states of Rio de Janeiro (RJ) and Espírito Santo (ES) are, in general, more susceptible. Consequently, areas with less susceptible populations were predominantly concentrated in the states of Minas Gerais (MG), Goiás (GO), and Bahia (BA), notably in the regions of Araxá (MG) and Luís Eduardo Magalhães (BA), which presented the highest LC_50_ values.

The coffee leaf miner populations exhibited only low levels of resistance (<7.48-fold). The resistance ratios (RR_50_), calculated using the Varre-Sai population as a reference, ranged from 1.0 to 7.48. The Araxá (MG) population showed the highest RR_50_ (7.48), followed by the Luís Eduardo Magalhães (BA) population (RR_50_ = 7.32), reflecting the considerable geographical variation in baseline susceptibility. The Rio Paranaíba 2 population (2.33 ± 0.49) exhibited the highest slope, while the Alfenas population (0.76 ± 0.11) showed one of the lowest values (Table 2).

**Fig. 3.**
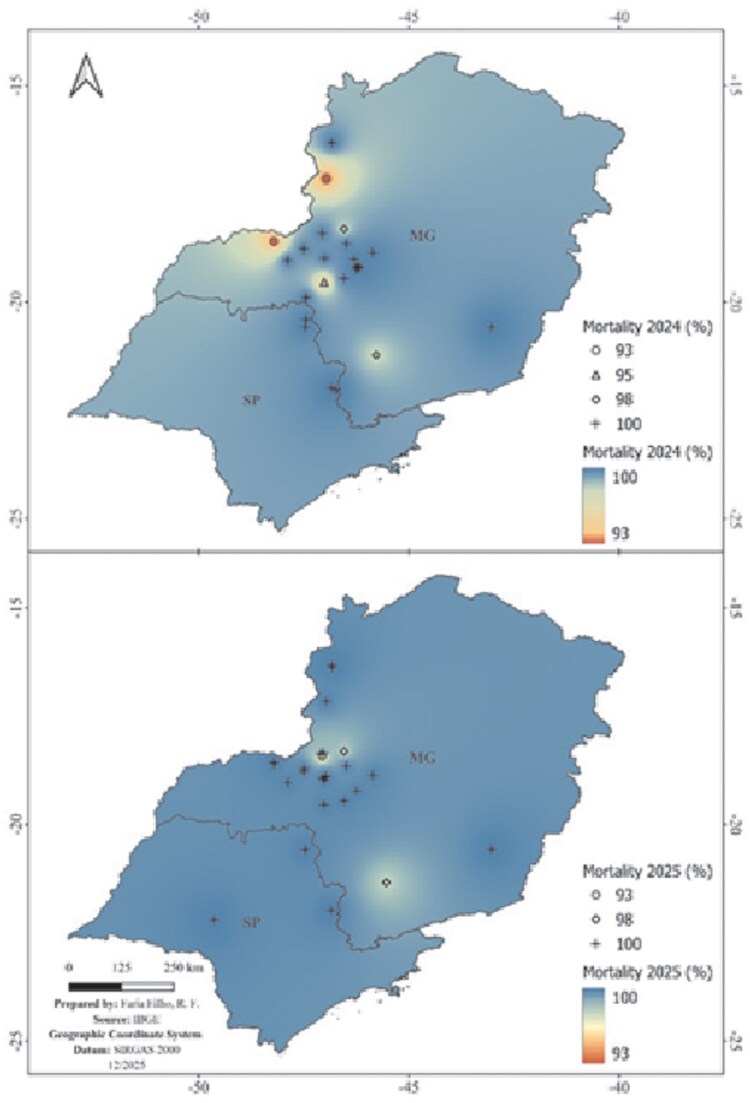
Spatial interpolation of *L. coffeella* mortality following application of the label dose of isocycloseram (150 mg a.i. L^−1^). The maps depict the geographical distribution of mortality (%) in the 2024 (upper map) and 2025 (lower map) crop seasons. Symbols (circle, triangle, diamond, and cross) indicate collection sites and the observed mortality percentage at each point. The color gradient (blue to red) indicates the mortality gradient (100% to 93%, respectively).

The study of CFL of the label dose (150 mg a.i. L^−1^) of isocycloseram was confirmed across all evaluated *L. coffeella* populations in the 2024 and 2025 crop seasons (Table 3). Observed mortality was consistently high, ranging from 93.0% to 100% in all monitored populations (Fig. 3). This result showed no significant deviation (P > 0.05) from the minimum threshold of agronomic efficacy of 80%. Notably, even the Araxá (MG) population, which showed the lowest susceptibility in the concentration-mortality bioassays (LC_50_ = 7.11 mg a.i. L^−1^), was effectively controlled, recording 95.0% mortality in 2024 and 100% in 2025. The spatial interpolation of mortality data (Fig. 3) revealed a wide area of maximum efficacy. However, in all cases, the CFL was negative [−16.3 to 25.0] for all tested populations.

**Table 3.** Estimated mortality of the isocycloseram label rate (and respective CFL) against the coffee leaf miner, *L. coffeella*, subjected to the insecticide label rate^a^

2024		2025	
Population	Mortality (%)	Population	Mortality (%)
**Araguari 1**	93.0 [−16.3]	Araguari 1	100 [−25.0]
**Araguari 2**	98.0 [−22.5]	Araguari 2	100 [−25.0]
**Araxá**	95.0 [−18.8]	Araxá	100 [−25.0]
**Campos Gerais**	98.0 [−22.5]	Coromandel 1	93.0 [−16.3]
**Carmo do Paranaíba**	100 [−25.0]	Garça	100 [−25.0]
**Coromandel 1**	100 [−25.0]	Monte Carmelo 1	100 [−25.0]
**Coromandel 2**	100 [−25.0]	Monte Carmelo 2	100 [−25.0]
**Coromandel 3**	98.0 [−22.5]	Patos de Minas	100 [−25.0]
**Coromandel 4**	100 [−25.0]	Patrocínio 1	100 [−25.0]
**Coromandel 5**	100 [−25.0]	Presidente Olegário	98.0 [−23]
**Cristais**	100 [−25.0]	Rio Paranaíba	100 [−25.0]
**Franca**	100 [−25.0]	Três Pontas	98.0 [−22.5]
**Guaraciaba**	100 [−25.0]	Unaí 1	100 [−25.0]
**Ibiá**	100 [−25.0]	Unaí 2	100 [−25.0]
**Indianópolis**	100 [−25.0]	Franca	100 [−25.0]
**Monte Carmelo 1**	100 [−25.0]	Guaraciaba	100 [−25.0]
**Monte Carmelo 2**	100 [−25.0]	Ibiá	100 [−25.0]
**Paracatu**	93.0 [−25.0]	Indianópolis	100 [−25.0]
**Patos de Minas**	100 [−25.0]	Coromandel 2	100 [−25.0]
**Patrocínio 1**	100 [−25.0]	Coromandel 3	100 [−25.0]
**Patrocínio 2**	100 [−25.0]	Paracatu	100 [−25.0]
**Patrocínio 3**	100 [−25.0]	São João da Boa Vista	100 [−25.0]
**Patrocínio 4**	95.0 [−25.0]	Tiros	100 [−25.0]
**Presidente Olegário 1**	98.0 [−25.0]	Patrocínio 2	100 [−25.0]
**Presidente Olegário 2**	100 [−25.0]	Patrocínio 3	100 [−25.0]
**Presidente Olegário 3**	100 [−25.0]	Patrocínio 4	100 [−25.0]
**Rio Paranaíba 1**	100 [−25.0]	–	–
**Rio Paranaíba 2**	100 [−25.0]	–	–
**Rio Paranaíba 3**	100 [−25.0]	–	–
**Rio Paranaíba 4**	100 [−25.0]	–	–
**Rio Paranaíba 5**	100 [−25.0]	–	–
**Sacramento**	100 [−25.0]	–	–
**São J. da Boa Vista 1**	100 [−25.0]	–	–
**São J. da Boa Vista 2**	100 [−25.0]	–	–
**Tiros**	100 [−25.0]	–	–
**Unaí**	100 [−25.0]	–	–

a Negative values within the bracket indicate absence of control failure. The CFL was determined following Guedes (2017).

## Discussion

The assessment of *L. coffeella* susceptibility to isocycloseram in the major coffee-growing regions of Brazil revealed favorable initial conditions, with no evidence of established field resistance to isocycloseram. However, the observed population variability highlights the importance of continuous monitoring to ensure the long-term sustainability of chemical control.

This variation also reflects differences in population homogeneity, with some populations potentially harboring more tolerant individuals, which could accelerate resistance evolution under selection pressure (Ffrench-Constant et al. 2004, Sparks and Nauen 2015). The spatial interpolation of this baseline variation reveals a mosaic of susceptibility across the study area, with no clear macroregional pattern. The most susceptible population (Varre-Sai/RJ) and the least susceptible ones (notably Araxá/MG and Luís Eduardo Magalhães/BA) represent extremes within a complex and spatially structured genetic landscape. This genetic variability is particularly concerning given the historical resistance of the coffee leaf miner to other insecticide classes. The documented varying levels of resistance to organophosphates, neonicotinoids, and the diamide chlorantraniliprole (Leite et al. 2020, 2021, Souza et al. 2025) demonstrate this pest’s adaptive capacity and serve as a warning for the imminent risk of resistance evolution to isocycloseram. This scenario contrasts with that observed for another key coffee pest, the coffee berry borer, *Hypothenemus hampei* (Ferrari) (Coleoptera: Curculionidae, Scolytinae), which exhibited relative uniformity in susceptibility to cyantraniliprole, a behavior attributed to the species’ low genetic diversity due to inbreeding (Costa et al. 2020). However, unlike the coffee berry borer, the coffee leaf miner is a sexually reproducing species with greater potential for gene flow, which may explain the greater natural variability in susceptibility documented and suggests an intrinsically higher risk for the rapid evolution of resistance.

This concern is heightened by the differences in the phenotypic homogeneity of the populations. While Rio Paranaíba 2 showed a steep slope, indicating a genetically more homogeneous population, Alfenas exhibited a shallow slope. This shallow slope suggests greater heterogeneity and the likely presence of naturally more tolerant individuals, which could be rapidly selected under insecticide pressure (Leite et al. 2021). The coexistence of populations with lower baseline susceptibility (Araxá and Luís Eduardo Magalhães) and greater phenotypic heterogeneity (Alfenas) identifies priority foci for a resistance monitoring program.

The results from the diagnostic concentration bioassays are consistent with this panorama, showing mortality greater than 90% in all populations tested in 2024 and 2025. These data reinforce the importance of the rational use of isocycloseram for resistance management and the implementation of tactics such as insecticide rotation.

However, the most relevant findings emerge from the efficacy tests of the field dose of isocycloseram, 150 mg a.i. L^−1^ (Syngenta 2024). The high mortality and absence of control failure across all populations, including Araxá, indicate that the recommended dose is currently effective. The spatial analysis of this efficacy confirms its uniformity, showing extensive areas of maximum control (100% mortality) across the evaluated regions. The label dose is approximately 1.4 times higher than the LC_99_ of the least susceptible population (Araxá), providing a safety margin that suppresses the documented baseline variation. Nevertheless, high insecticide doses, while effective, exert strong selection pressure on the population, favoring the survival of more resistant individuals over susceptible ones (Helps et al. 2017). Thus, the current effectiveness of the recommended dose should not be interpreted as a substitute for resistance management strategies, as it may accelerate the fixation of resistance alleles in heterogeneous populations. Evaluating control failure was also important to confirm that field populations were generally susceptible to isocycloseram, providing support for the establishment of reliable baseline dose–response parameters.

This comprehensive study opens new avenues for future research. The biochemical and genetic mechanisms underlying the observed baseline variation remain unknown. It is necessary to investigate whether the differences in LC_50_ are mediated by enzymatic metabolism, such as cytochrome P450 monooxygenases, esterases, or glutathione S-transferases, or by polymorphisms at the target site (Hawkins et al. 2019, Liu et al. 2022). The historical selection pressure from other insecticide classes in Brazilian coffee crops (Dantas et al. 2021) may have selected for generalist detoxification mechanisms (Feyereisen 2015) that, while not currently conferring evident cross-resistance, could facilitate the evolution of specific resistance to isocycloseram.

Our study established the baseline susceptibility of *L. coffeella* to isocycloseram, determining the diagnostic concentrations for future monitoring as LC_50_ = 1.23, LC_80_ = 12.06, and LC_99_ = 39.76 mg a.i. L^−1^. The results confirm that isocycloseram is currently highly effective, with the field rate providing over 93% mortality and zero control failure risk against contemporary populations. Nevertheless, the 7.5-fold natural variation in baseline susceptibility serves as a critical warning, revealing a high genetic potential and a tangible risk for resistance evolution. Therefore, the long-term sustainability of this insecticide is contingent upon the immediate implementation of a continuous monitoring program, using the established baseline doses, coupled with the rigorous adoption of resistance management strategies, primarily rotation with insecticides from distinct mode of action groups (Benvenuto et al. 2024).

## Data Availability

The datasets generated and/or analyzed during the current study are available from the corresponding author on reasonable request.
